# Molecular Insight of Plants Response to Drought Stress: Perspectives and New Insights towards Food Security

**DOI:** 10.3390/ijms25094988

**Published:** 2024-05-03

**Authors:** Isabel Marques, Honghong Hu

**Affiliations:** 1 Forest Research Centre, Associate Laboratory TERRA, School of Agriculture, University of Lisbon, 1349-017 Lisbon, Portugal; 2 Key Laboratory of Crop Genetic Improvement, College of Life Science and Technology, Huazhong Agricultural University, Wuhan 430070, China

## 1. Plant Molecular Responses to Cope with Drought

In the face of climate-induced challenges, understanding the intricate molecular mechanisms underlying drought tolerance in plants has become imperative [1]. Unraveling these mechanisms not only offers insights into plant resilience but also opens avenues for enhancing crop productivity and environmental conservation [2,3]. Armed with this knowledge, scientists can employ innovative biotechnological approaches to develop drought-tolerant crop varieties [4,5]. In this context, this Special Issue covers applied research aimed at understanding the molecular mechanisms associated with plant responses to drought. By identifying genetic variants associated with drought resilience, we can elucidate the underlying molecular mechanisms and facilitate the breeding development of drought-tolerant crop varieties.

## 2. List of Contributions

1. Liu, W.; Wang, T.; Wang, Y.; Liang, X.; Han, J.; Hou, R.; Han, D. The Transcription Factor MbWRKY46 in *Malus baccata* (L.) Borkh Mediate Cold and Drought Stress Responses. *Int. J. Mol. Sci.* **2023**, *24*, 12468. https://doi.org/10.3390/ijms241512468.
2. Baek, D.; Cho, H.M.; Cha, Y.J.; Jin, B.J.; Lee, S.H.; Park, M.S.; Chun, H.J.; Kim, M.C. Soybean Calmodulin-Binding Transcription Activators, *GmCAMTA2* and *GmCAMTA8*, Coordinate the Circadian Regulation of Developmental Processes and Drought Stress Responses. *Int. J. Mol. Sci.* **2023**, *24*, 11477. https://doi.org/10.3390/ijms241411477.
3. Jiao, P.; Liang, Y.; Chen, S.; Yuan, Y.; Chen, Y.; Hu, H. *Bna.EPF2* Enhances Drought Tolerance by Regulating Stomatal Development and Stomatal Size in *Brassica napus*. *Int. J. Mol. Sci.* **2023**, *24*, 8007. https://doi.org/10.3390/ijms24098007.
4. Pecetti, L.; Annicchiarico, P.; Crosta, M.; Notario, T.; Ferrari, B.; Nazzicari, N. White Lupin Drought Tolerance: Genetic Variation, Trait Genetic Architecture, and Genome-Enabled Prediction. *Int. J. Mol. Sci.* **2023**, *24*, 2351. https://doi.org/10.3390/ijms24032351.
5. Wu, B.; Chen, S.; Cheng, S.; Li, C.; Li, S.; Chen, J.; Zha, W.; Liu, K.; Xu, H.; Li, P.; et al. Transcriptome Analysis Revealed the Dynamic and Rapid Transcriptional Reprogramming Involved in Cold Stress and Related Core Genes in the Rice Seedling Stage. *Int. J. Mol. Sci.* **2023**, *24*, 1914. https://doi.org/10.3390/ijms24031914.
6. Liang, B.; Wan, S.; Ma, Q.; Yang, L.; Hu, W.; Kuang, L.; Xie, J.; Huang, Y.; Liu, D.; Liu, Y. A Novel bHLH Transcription Factor *PtrbHLH66* from Trifoliate Orange Positively Regulates Plant Drought Tolerance by Mediating Root Growth and ROS Scavenging. *Int. J. Mol. Sci.* **2022**, *23*, 15053. https://doi.org/10.3390/ijms232315053.
7. Melton, A.E.; Galla, S.J.; Dumaguit, C.D.C.; Wojahn, J.M.A.; Novak, S.; Serpe, M.; Martinez, P.; Buerki, S. Meta-Analysis Reveals Challenges and Gaps for Genome-to-Phenome Research Underpinning Plant Drought Response. *Int. J. Mol. Sci.* **2022**, *23*, 12297. https://doi.org/10.3390/ijms232012297.

Contribution 1 isolated and cloned a new *WRKY TF* gene, *MbWRKY46*, from *Malus baccata* (L.) Borkh. and transformed it into *Arabidopsis* plants to obtain transgenic lines. The study revealed that the expression of *MbWRKY46* was tissue-specific, with the highest expression level being found in roots and old leaves. Cold, heat, high salinity, drought, and ABA stimulated the expression of *MdWRKY46*. Its overexpression activated the expression of downstream target genes through binding to CBF/DREB or ABA-dependent pathways to improve the cold and drought resistance of transgenic plants. Overall, this study provides new information about the role of the *WRKY TF* family in *Malus baccata* (L.) Borkh. in stress resistance.

Contribution 2 preliminary explored the roles of Calmodulin-Binding Transcription Activators (*GmCAMTA*) in soybean (*Glycine max* (L.) Merr. cv. Williams 82) development processes and stress tolerance responses. A total of 15 *GmCAMTAs* genes were significantly expressed with exposure to light, and several were induced by stress conditions. The results also indicated that these *GmCAMTA* genes are expressed in different plant tissues. To further determine the functional roles of these genes, the authors overexpressed *GmCAMTA2* and *GmCAMTA8* in *Arabidopsis* plants. Some stress-responsive genes were downregulated in the *GmCAMTA2-OX* and *GmCAMTA8-OX* plants under drought conditions compared to wild-type plants. These results indicate that *GmCAMTA2* and *GmCAMTA8* may act as negative regulators in drought stress responses.

Contribution 3 identified the role of *Bna.EPF2*, an epidermal patterning factor (*EPF*), in drought responses in *Brassica napus* L. (ecotype Westar). *Bna.EPF2* overexpression in rapeseed lines showed a reduction in stomatal density. This resulted in a higher WUE, a lower transpiration rate, and stomatal conductance under normal growth conditions and increased drought tolerance compared to wild-type plants under water-limited conditions. Nevertheless, *Bna.EPF2* overexpression did not affect major agronomic traits such as plant height, the length of main inflorescence, the number of siliques on main inflorescence, the number of first branches, 1000-seed weight, or seed yield per plant. These results demonstrated that the overexpression of *Bna.EPF2* affected leaf transpiration by regulating stomatal density and dimensions in *B. napus*, further suggesting the potential role of this gene in enhancing drought tolerance in plants.

Contribution 4 studied a genetically broad population of 138 lines of white lupins (*Lupinus albus* L.) to investigate the phenotypic variation and genotype × environment interaction (GEI) for grain yield and other traits across drought-prone and moisture-favorable managed environments. A genome-wide association study revealed several significant SNPs linked to grain yield and the onset of flowering, which differed across conditions. Overall, this study revealed the presence of heritable, polygenic genetic variation involved in drought tolerance that could be exploited for crop selection and improvement.

Contribution 5 explored the transcriptional regulation mechanism and the reason for the phenotypic divergence found in two rice lines (*Oryza sativa* L.) in response to cold stress. Although two different rice lines were used, the cold-sensitive ZL31 and the cold-resistant Towada, the results showed that a rapid and high-amplitude transcriptional reprogramming occurred at the early stage of cold treatment in both lines. Responses to cold stress involved environmental adaptation, signal transduction, metabolic activity, photosynthetic changes, as well as the MAPK signaling pathway. Additionally, authors identified four core genes, *OsWRKY24*, *OsCAT2*, *OsJAZ9*, and *OsRR6*, as potential candidates affecting the cold sensitivity of rice lines.

Contribution 6 isolated and characterized a drought- and ABA-responsive gene, PtrbHLH66, from trifoliate orange (*Poncirus trifoliata* (L.) Raf.). Ectopic expression and gene-silencing assays showed that *PtrbHLH66* positively regulated the expression levels of genes involved in ABA biosynthesis, proline biosynthesis, ROS scavenging, and drought response, including the dehydration-responsive element-binding genes *DREB1A*, *DREB2A*, and *DREB3* and the early responsive to dehydration 1 gene, *ERD1*. These results provided new insight into the molecular mechanism of citrus’s response to drought stress and provided a novel candidate gene for drought-tolerant breeding efforts.

Contribution 7 developed a pipeline and performed a meta-analysis to identify genes involved in drought-stress responses. Several genes contribute to multiple drought response strategies such as *oasC* (O-acetylserin (thiol) lyase isoform C), *cysC* (cysteine synthase C1), *NSY* (chloroplastic neoxanthin synthase), *CITRX* (Cf-9-interacting thioredoxin), *NAC* (NAM, ATAF, and CUC transcription factors), *WRKY* (WRKY-domain containing transcription factors), *SODCC.1* (superoxide dismutase [Cu-Zn] 1), *MYB* (MYB DNA-binding domain-containing transcription factors), *PLA3* (probable glutamate carboxypeptidase), and *LEA* (late embryogenesis abundant protein). Authors also identified a bias towards model plants. Nevertheless, several species occurring in hyper-arid ecosystems have been sequenced and would be a good choice for future research on drought responses.

## 3. Challenges and New Perspectives

Although numerous genes and pathways associated with drought responses have been identified, many issues remain challenging:

Dynamic Regulatory Networks: Drought stress triggers dynamic changes in gene expression and protein activity that change over time and across different tissues [6]. Understanding and integrating the temporal and spatial dynamics of regulatory networks will be crucial for unraveling the complexity of plant drought responses.

Epigenetic Regulation: Epigenetic mechanisms, including DNA methylation, histone modifications, and small RNA-mediated gene silencing, play pivotal roles in modulating plant responses [7,8]. However, our understanding of how they contribute to drought tolerance remains scarce. Further research is needed to elucidate the functional significance of epigenetic changes and their interplay with genetic pathways in shaping plant adaptation to water scarcity.

Environmental Interactions: Drought often interacts with other environmental stresses, such as heat, cold, salinity, and nutrient deficiency, sometimes with very different outcomes [2,9]. Investigating the crosstalk between drought-responsive pathways and other stress signaling networks will provide insights into the complex regulatory mechanisms governing plant stress responses.

Translational Research: Bridging the gap between basic research and practical applications is essential for harnessing molecular insights into enhancements in crop drought tolerance [10]. Translational research efforts should focus on developing novel breeding strategies, genetic engineering techniques, and precision agriculture approaches tailored to specific cropping systems and environmental conditions. Furthermore, ensuring equitable access to drought-tolerant crop varieties and technologies is crucial for addressing global food security challenges in the face of climate change.

In conclusion, while significant progress has been made in unraveling the molecular mechanisms of plant drought tolerance, continued interdisciplinary research efforts are needed to address the remaining challenges. By advancing our understanding of plant responses to drought at the molecular level, we can pave the way for sustainable agricultural practices and resilient food systems in a changing climate.
